# Triple Labeling Resolves a GPCR Intermediate State by Using Three-Color Single Molecule FRET

**DOI:** 10.1021/jacs.4c18364

**Published:** 2025-05-15

**Authors:** Léo Bonhomme, Ecenaz Bilgen, Caroline Clerté, Jean-Philippe Pin, Philippe Rondard, Emmanuel Margeat, Don C. Lamb, Robert B. Quast

**Affiliations:** † Centre de Biologie Structurale (CBS), University of Montpellier, CNRS, INSERM, Montpellier 34090, France; ‡ Department of Chemistry and Center for Nanoscience, Ludwig-Maximilians-Universität München (LMU), Munich 81377, Germany; § Institut de Génomique Fonctionnelle (IGF), University of Montpellier, CNRS, INSERM, Montpellier 34090, France

## Abstract

The correlation of individual conformational changes in dynamic protein complexes remains challenging as most structural methods rely on averaged information over a large number of molecules. Single molecule FRET is a powerful tool for monitoring such conformational changes. When performed using three distinct probes, it enables the correlation of domain movements by providing up to three simultaneous distance measurements with high temporal resolution. Nevertheless, a major challenge lies in the site-specific attachment of three probes to unique positions within the target protein. Here, we propose an orthogonal triple-labeling strategy that is not compromised by native, reactive amino acid functionalities. It combines genetic code expansion and bioorthogonal labeling of two different noncanonical amino acids with an enzymatic self-labeling SNAP tag. We demonstrate its application by establishment of a 3-color sensor on the human metabotropic glutamate receptor 2, a dimeric, multidomain G protein-coupled neuroreceptor, and describe a previously unknown conformational intermediate state using 3-color single molecule FRET.

## Graphical abstract

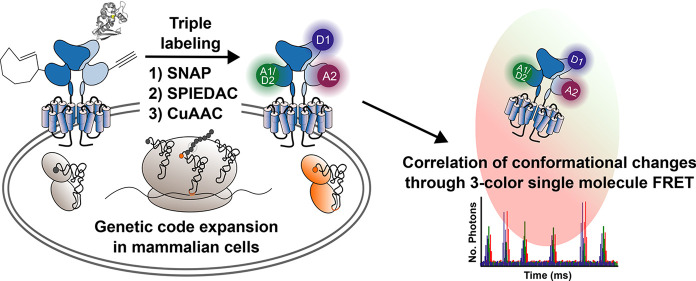

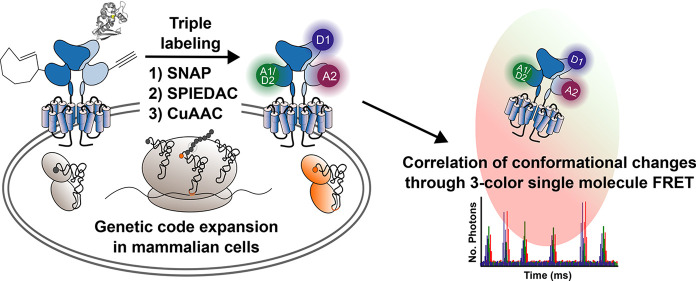

## Introduction

Multidomain protein complexes experience concerted conformational changes to exert their specific functions. However, a quantitative assessment of the visited conformational states and transition time scales remains challenging. Förster resonance energy transfer (FRET) is a powerful tool to derive distance information on the molecular scale from single molecules and allows one to capture transient intermediates that may be obscured by ensemble averaging.^1^ Single molecule FRET (smFRET) experiments have become recognized as a powerful approach for determining distances between two probes attached to proteins.^2^ However, it is not possible to extract correlated motions between individual domains from such unidimensional information. To address this, smFRET employing three, spectrally separated fluorophores, can be used to simultaneously monitor and correlate three distances on a single protein or complex. This opens the possibility of identifying and quantifying transient intermediate populations in dynamic biomolecular systems.^3^ However, the major challenge in 3-color smFRET remains the efficient and specific conjugation of three fluorophores to selected positions on the target protein. Indeed, stochastic labeling of three or more positions using the same chemistry complicates the interpretation of the FRET histograms due to the appearance of several FRET populations between undefined labeling positions, and thus impairs correct assignment of measured FRET efficiencies to the distance changes of interest. Previous solutions to this difficulty employed cysteine-maleimide chemistry in combination with other labeling approaches. For instance, Voss et al. combined labeling of an exposed cysteine in a truncated Rab1b GTPase N-terminally fused to a fluorescent protein with C-terminal oxime ligation after conversion of an intein tag to an oxyamine protein derivative.^4^ As an alternative, Yoo et al. first conjugated dyes to a cysteine and a site-specifically incorporated noncanonical amino acid (ncAA) on the designer protein α3D and then introduced an additional, reactive cysteine through C-terminal, sortase-mediated ligation.^5^ While these approaches enable site-specific triple labeling, both rely on additional C-terminal transformations before the third dye can be attached. Furthermore, fluorescent proteins are today rarely employed in quantitative smFRET measurements due to their low brightness and photostability. Stochastic labeling of two cysteines combined with a ncAA leads to only two labeling subpopulations and has also been employed for 3-color smFRET on the FG-rich domain of the yeast nucleoporin 49 and different heat shock protein 70 chaperones.^6–8^ The stochastic labeling can be accounted for in the analysis but the labeling efficiency of the stochastic labels needs to be known or estimated, and the correlation one wishes to investigate gets masked within the data. Specificity can be promoted when using two cysteines by blocking the accessibility,^9^ allowing specific labeling of three colors when used in combination with a ncAA, as has been done for the maltose binding protein,^10^ or by taking advantage of differing cysteine reaction kinetics, as was done for cytolysin A.^11^ While the above-mentioned approaches ingenuously tackle the challenge for their respective systems, they are not generalizable to all systems. In addition, they exclusively represent examples of soluble proteins expressed in bacteria. Thus, there is still a need for more generalizable strategies that can be applied to a broader spectrum of targets including eukaryotic membrane proteins and where the invasiveness of the labeling procedures is minimized.

In particular, the functional importance of native cysteines in many proteins strongly limits their use to proteins that do not contain cysteines or a small number of nonfunctionally relevant cysteines.^12^ An attractive alternative to cysteines for labeling are ncAAs, which barely exceed the size of proteinogenic amino acids, and equip proteins with chemical handles that can subsequently be used to conjugate the desired organic dyes in a site-specific and bioorthogonal manner.^13,14^ The most prominent examples of reactive ncAAs comprise derivatives of tyrosine/phenylalanine and pyrrolysine to incorporate azides as well as terminal and strained alkenes and alkynes. These moieties have found notable applications because (i) robust and efficient tRNA/synthetase pairs have been evolved for orthogonal use in both bacteria and mammalian cells, (ii) bioorthogonal reactions such as the Staudinger ligation, copper-catalyzed azide–alkyne cycloaddition (CuAAC), strain-promoted azide–alkyne cycloaddition (SPAAC) and strain-promoted inverse electron-demand Diels–Alder cycloaddition (SPIEDAC) provide good reaction kinetics and proceed with high selectivity, and (iv) reactive dye derivatives are commercially available.

The selective incorporation of ncAAs at desired positions within the protein-of-interest can be achieved through the suppression of premature stop codons, which are easily introduced by site-directed mutagenesis of the target gene. Incorporation *in cellulo* is mediated by evolved aminoacyl-tRNA synthetase/tRNA pairs that behave orthogonal to the host protein synthesis machinery. Thanks to the orthogonality between several of these pairs, two^15,16^ and even three distinct ncAAs^17^ have been successfully incorporated into proteins in mammalian cells in response to *Amber* TAG, *Ochre* TAA and *Opal* TGA stop codons. However, the use of multiple stop codons comes at the cost of reduced protein yields due to the required cotransfection of multiple tRNA/synthetase pairs and competition of stop codon suppression with translation termination, which has so far impaired triple ncAA labeling for FRET studies based on mammalian expression systems.

Regarding the reaction chemistries, a major limitation of the CuAAC reaction arises from the cytotoxicity of the copper catalyst and reaction byproducts that can harm proteins.^18^ The use of picolyl-azides allows reducing copper concentrations while maintaining fast reaction kinetics.^19^ Accordingly, we recently established live-cell compatible labeling conditions and developed a set of conformational smFRET sensors for the metabotropic glutamate receptor 2 (mGlu2) through incorporation of propargyl-l-phenylalanine (PrF) in response to TAG in mammalian HEK293T cells.^20^ The mGlu2 receptor is a class C G protein-coupled receptor, essential for the regulation of neuronal excitability and synaptic transmission.^21^ Cryo-electron microscopy structures of this dimeric receptor (stabilized by a native disulfide bridge between the two protomers, Figure 1) point at a transition from an inactive, resting open (R_O_) state to an active, closed state (A_C_) during their activation.^22–24^ This involves a ligand-induced closure of the Venus flytrap (VFT) domains (open o → c closed transition), a reorientation of the two subunits relative to each other, which bring the lower lobes of the VFT domains in closer proximity (resting R → A active transition), and changes to the interface of adjacent 7 transmembrane (7TM) domains (Figure 1). Using a 2-color smFRET sensor to probe the o → c transition, we recently revealed that saturating concentrations of the natural full agonist glutamate led to an efficient closure of the VFT domains.^20^ However, when monitoring the R → A transition, we discovered the coexistence of at least two states corresponding to the resting and active orientations. These data suggest an agonist-induced dynamic equilibrium between at least two states including an intermediate state between the R_O_ and the A_C_ state (Figure 1). However, a direct and simultaneous correlation of VFT domain closure and reorientation would be necessary to identify and characterize this intermediate state.

**Figure 1:**
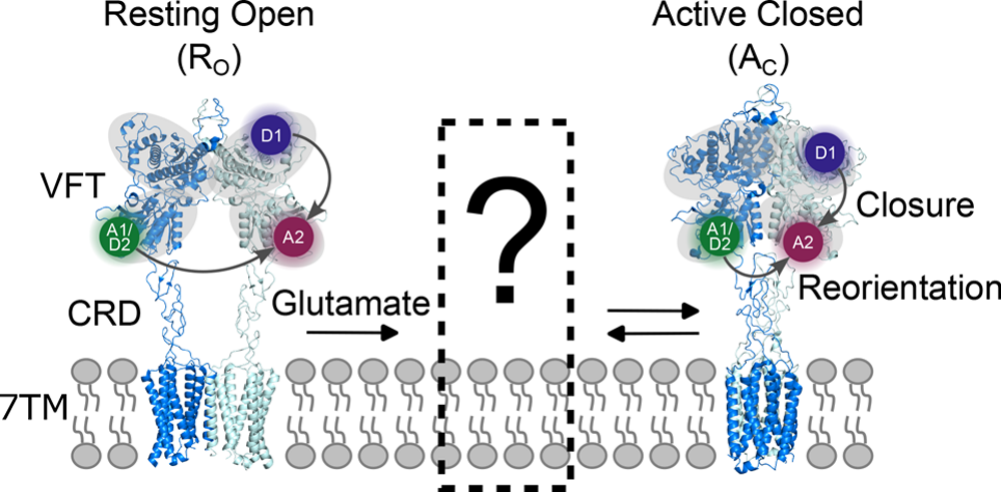
Major conformational changes during mGlu2 activation. Cryo-EM structures of the mGlu2 receptor (left) in the resting open (*R*_O_) state with Venus flytrap (VFT) domains open, the lower lobes and cysteine-rich domains (CRDs) separated, and an interface mediated by the transmembrane domain helices IV (PDB 7EPA) and (right) in the active closed (*A*_C_) state with the VFT domains closed, the lower lobes and CRDs in closer proximity and a slightly twisted dimer interface mediated by 7TM helices VI and VII (PDB 7EPB). The upper and lower lobes of the VFT domains are shaded in gray. The labeling positions used to establish a 3-color smFRET sensor are indicated by colored circles (Donor 1 (D1): purple; Acceptor 1/Donor 2 (A1/D2): green; Acceptor 2 (A2): red).

To characterize this potential intermediate state, we established an orthogonal, site-specific triple labeling strategy based on the incorporation of two different ncAAs and a SNAP-tag. We chose SNAP-tag labeling for the first donor (Figure 1, D1) as it does not affect expression yields and labeling is fast and selective. Furthermore, we used a combination of SPIEDAC between a trans-cyclooctene lysine (TCOK) with a tetrazine dye derivative to attach the first acceptor/second donor (Figure 1, A1/D2) and CuAAC between an incorporated PrF with a picolyl-azide dye derivative to attach the second acceptor (Figure 1, A2). Using the resultant 3-color sensor, we simultaneously measured FRET for single dimers between all dye pairs and investigated the upper and lower lobes (Figure 1, D1 → A1/D2) within a single protomer to follow VFT domain closure and the lower lobes between the two protomers (Figure 1, A1/D2 → A2) to follow their reorientation. This allowed us to uncover a previously uncharacterized intermediate state in which the VFT domain is only slightly closed, while the lower lobes remain in the resting orientation during glutamate activation of the mGlu2 receptor.

## Experimental Section

### Chemicals

Aminoguanidine hydrochloride, copper­(II) sulfate, (+)-sodium l-ascorbate, and l-glutamate were purchased from Sigma-Aldrich (St. Louis, MO, USA) unless stated otherwise. SNAP-Surface Alexa Fluor 488 was purchased from NEB (Evry, France). The SNAP Cy3B and Lumi4-Tb were purchased from Revvity (Codolet, France). O-Propargyl-l-tyrosine hydrochloride (PrF) was obtained from Iris Biotech GmbH (Marktredwitz, Germany) and trans-Cyclooct-2-en-l-Lysine TCO*A (TCOK) from Sirius Fine Chemicals GmbH (Bremen, Germany). 2-(4-((bis­((1-(*tert*-butyl)-1H-1,2,3-triazol-4-yl)­methyl)­amino)­methyl)-1H-1,2,3-triazol-1-yl)­acetic acid (BTTAA), Atto488 tetrazine, AF647-picolyl-azide and AF546-picolyl-azide were purchased from Jena Bioscience (Jena, Germany). Cy3B tetrazine was obtained from AAT Bioquest (Pleasanton, USA). Lauryl maltose neopentyl glycol (LMNG) and cholesteryl hemisuccinate tris salt (CHS) were purchased from Anatrace (through CliniSciences, France). Glyco-diosgenin (GDN) was purchased from Avanti Polar Lipids through Merck.

### DNA Constructs

The pcDNA plasmid encoding human mGlu2 with N-terminal FLAG (Sigma-Aldrich) and SNAP-tags (New England Biolabs) was a gift from Revvity (Codolet, France).

We used the engineered GABA_B_ C-terminal tails, which promote the selective presentation of the desired C1 and C2 heterodimers at the cell surface for specific labeling.^20,25^ These were obtained using restriction enzymes from the pRK5 plasmids encoding for rat mGlu2 (rat-mGluR2-C1KKXX and rat-mGluR2-C2KKXX) as described in.^26^ They were purified by agarose electrophoresis and ligated in-frame into the human mGlu2 gene cut using the same restriction enzymes.

Premature stop codons (A248TAG, A248TAA, S246TAA, A256TAA, L258TAA, R284TAA, G306TAA, A310TAA, A311TAA, R358TAA) were introduced using the QuikChange Lightning Site-directed Mutagenesis kit from Agilent Technologies (Santa Clara, CA, USA) according to the manufacturer’s protocol. Final stop codons were changed from TAA to TGA to circumvent additional incorporation of TCOK at the receptor’s C-terminus following the same protocol. In selected constructs, the N-terminal FLAG and SNAP-tags were removed to generate dimers without SNAP-tag or SNAP-tags only within a single protomer using the In-Fusion HD cloning kit (Takara Bio Europe) according to the manufacturer’s protocol. An overview of all mGlu2 constructs can be found in Table S1 together with a reference to their respective data Figures.

The pNEU-hMbPylRS­(AF)-4xU6M15 plasmid, containing a single copy of the Y271A, Y349F mutant Methanosarcina barkeri pyrolysyl-tRNA synthetase codon-optimized for humans and four copies of the Methanosarcina mazei derived tRNA_UUA_, used for incorporation of TCOK in response to TAA^27^ and the pIRE4-PGK-ePrFRS plasmid, harboring a single copy of the Y37T, D182S, F183A, D256R mutant Escherichia coli tyrosyl-tRNA synthetase and four copies of the Bacillus stearothermophilus derived tRNA_CUA_ used for incorporation of PrF in response to TAG,^20,27^ were a kind gift of I. Coin (Universität Leipzig, Germany).

### Protein Expression, Labeling and Purification

All expressions were carried out using adherent HEK293T cells (American Type Culture Collection CRL-3216, LGC Standards S.a.r.l., France) cultured in Dulbecco’s modified Eagle’s medium (DMEM; Thermo Fisher Scientific) supplemented with 10% fetal bovine serum (FBS; Sigma-Aldrich) at 37 °C with 5% CO_2_. To improve adherence, all culture vessels were treated with a 28.5 μg/μL solution of poly-l-ornithine-hydrobromide (Sigma) in DPBS at 37 °C for 15 min followed by a single wash using DPBS before cell plating. HEK cells were transfected using JetPrime transfection reagent (Polyplus-transfection SA, Illkirch-Graffenstaden, France).

For the screening of TCOK incorporation in response to TAA at positions within the lower lobe and evaluation of the selective incorporation of PrF in response to TAG and TCOK in response to TAA, HEK293T cells were cultured in 96-well F-bottom black plates (Greiner Bio-One). A total of 75,000 cells were seeded in 100 μL of DMEM per well 17 h before transfection. Cells were cotransfected with 125 ng of vectors coding for FLAG-SNAP-mGlu2 containing the respective premature stop codon mutations and 125 ng of pNEU-hMbPylRS­(AF)-4xU6M15 (lower lobe screening) or 62.5 ng pNEU-hMbPylRS­(AF)-4xU6M15 and 62.5 ng pIRE4-PGK-ePrFRS (selective incorporation assay) per well in 30 μL of JetPrime buffer (Polyplus). To test for unspecific dye labeling and for autofluorescence correction, the receptor DNA was replaced by an empty pRK6 vector. A total of 0.5 μL of JetPrime Reagent per well was added and incubated for 25 min at room temperature. Last, the transfection mixtures were added to the cells and incubated at 37 °C. After 6 h, medium was replaced with 100 μL fresh DMEM per well, supplemented with either 0.2 mM TCOK or 0.3 mM PrF. The medium was further exchanged with fresh ncAA-containing medium after 24 and 48 h. 72 h after transfection, SNAP-tag labeling was performed on adherent cells at 37 °C and 5% CO_2_ for 2 h using a final concentration of 100 nM SNAP-Lumi4-Tb (PerkinElmer, Codolet, France) in Gibco DMEM GlutaMax without phenol red, supplemented with GlutaMAX and pyruvate (Thermo Fisher Scientific, France). Following labeling, excess dye was removed by three cycles of washing with DPBS without Ca^2+^ and Mg^2+^ (Thermo Fischer Scientific, France) at ambient temperature. Time-resolved Lumi4-Tb fluorescence detection on living cells was performed in 50 μL acquisition buffer (20 mM HEPES pH 7.4, 118 mM NaCl, 1.2 mM KH_2_PO_4_, 1.2 mM MgSO_4_, 4.7 mM KCl and 1.8 mM CaCl_2_) on an Infinite F500 plate reader (Tecan) with excitation at 320/25 nm and emission collection at 635/35 nm. The signal was integrated after a lag time of 100 μs for 400 μs, corrected for unspecific labeling and autofluorescence, and plotted using Prism 7.05 (GraphPad).

For the labeling specificity assay, HEK293T cells were cultured in a standard flat 6-well TC-plate (Sarstedt). A total of 1,200,000 cells were seeded in 2 mL of DMEM per well 17 h before transfection. For the PrF condition, cells were transfected with vectors coding for mGlu2-C2KKXX, mGlu2-A248TAG-C1KKXX, pIRE4-PGK-ePrFRS and pNEU-hMbPylRS­(AF)-4xU6M15 in a 1:3:2:2 ratio using 2 μg of DNA in 200 μL of JetPrime buffer (Polyplus) per well. For the TCOK condition, cells were transfected with vectors coding for mGlu2-C1KKXX, mGlu2-L258TAA-C2KKXX, pIRE4-PGK-ePrFRS and pNEU-hMbPylRS­(AF)-4xU6M15 in a 1:3:2:2 ratio using 2 μg of DNA per well in 200 μL of JetPrime buffer (Polyplus) per well. For the SNAP condition, cells were transfected with vectors coding for FLAG-SNAP-mGlu2-C1KKXX, mGlu2-C2KKXX, pIRE4-PGK-ePrFRS and pNEU-hMbPylRS­(AF)-4xU6M15 in a 1:1:1:1 ratio using 2 μg of DNA in 200 μL of JetPrime buffer (Polyplus) per well. For the triple labeling condition, cells were transfected with vectors coding for SNAP-mGlu2-A248TAG-C1KKXX, mGlu2-L258TAA-C2KKXX, pIRE4-PGK-ePrFRS and pNEU-hMbPylRS­(AF)-4xU6M15 in a 1:1:1:1 ratio using 2 μg of DNA in 200 μL of JetPrime buffer (Polyplus) per well. Four μL of JetPrime Reagent per well were added to the transfection mixtures and incubated for 25 min at room temperature. Last, the transfection mixtures were added to the cells and incubated at 37 °C for 6 h. After 6h, medium was replaced with 2 mL fresh DMEM supplemented with 0.2 mM TCOK and 0.3 mM PrF. The medium was further exchanged with fresh ncAA-containing medium after 24 and 48 h. 72h after transfection, SNAP-tag labeling was performed on adherent cells at 37 °C and 5% CO_2_ for 2h using a final concentration of 600 nM SNAP-Cy3B. Excess dye was removed by three cycles of washing with DPBS without Ca^2+^ and Mg^2+^ at RT. TCOK labeling was performed right after SNAP-tag labeling using a final concentration of 1 μM Cy3B tetrazine in acquisition buffer incubated for 15 min at 37 °C. Excess dye was removed by three cycles of washing with DPBS at room temperature. PrF labeling was performed last using a final concentration of 10 μM AF546-picolyl-azide, 1.5 mM Aminoguanidine, 1.98 mM BTTAA, 0.36 mM CuS0_4_ and 2 mM Na-Ascorbate in acquisition buffer for 15 min at 37 °C. Excess dye was removed by three cycles of washing with DPBS. For crude membrane fractions preparation, adherent cells were detached mechanically using a cell scraper in DPBS and collected at 1000 × *g* and 22 °C for 5 min. Subsequently, cells were resuspended in cold hypotonic lysis buffer (10 mM HEPES pH 7.4 and cOmplete protease inhibitor, Roche), frozen, and stored at −80 °C. After thawing, cells were passed through a 200 μL pipet tip 30 times on ice. After two rounds of centrifugation at 500 × *g* and 4 °C for 5 min, the supernatant was centrifuged at 21,000 × *g* and 4 °C for 30 min to collect crude membranes. The pellets were washed once with acquisition buffer, flash frozen in liquid N_2_ and stored at −80 °C until solubilization. Receptors were solubilized using 10 μL of acquisition buffer containing 1% LMNG (w/v) and 0.1% CHS Tris (w/v) per membrane fraction (corresponding to cells cultured in one well of a six-well plate) for 15 min on ice. Subsequently, the solubilization mixture was centrifuged for 10 min at 4 °C and 21,000 × *g*. The supernatant was mixed with SDS-loading dye without bromophenol blue and separated by SDS-PAGE (NuPAGE 4 to 12% Bis–Tris gels, Thermo Fisher) before in-gel fluorescence was detected using a fluorescence scanner (Typhoon FLA 9000, GE Healthcare) with 532 nm excitation and a 575 nm long-pass detection filter.

The samples for 2-color smFRET experiments were prepared in 6 well plates as described above using the following ratios at a total of 2 μg DNA per well: FLAG-SNAP-mGlu2-A248TAG-C1KKXX, mGlu2-C2KKXX, pIRE4-PGK-ePrFRS 2:1:3; FLAG-SNAP-mGlu2-A248TAG-C1KKXX, mGlu2-A258TAA-C2KKXX, pIRE4-PGK-ePrFRS, pNEU-hMbPylRS­(AF)-4xU6M15 1:1:1:1; mGlu2-A248TAG-R358TAA-C1KKXX, pIRE4-PGK-ePrFRS, pNEU-hMbPylRS­(AF)-4xU6M15 1:4:2.5:2.5. After solubilization, the supernatants were mixed with 90 μL of acquisition buffer containing 0.11% GDN (w/v). The diluted samples were then passed through Zeba Spin desalting columns (0.5 mL, 7 kDa cutoff; Thermo Fisher Scientific, France) equilibrated in acquisition buffer containing 0.005% LMNG (w/v), 0.0005% CHS Tris (w/v) and 0.005% GDN (w/v). Finally, samples were diluted 1:40 in acquisition buffer and further diluted in acquisition buffer containing 0.0025% LMNG (w/v), 0.00025% CHS Tris (w/v) and 0.0025% GDN (w/v) to obtain single molecule-compatible concentrations while maintaining the detergents above the critical micelle concentration.

For 3-color smFRET experiments, HEK293T cells were cultured in T-75 surface flasks: Cell+, 2-position screw cap flasks (Sarstedt). A total of 10,000,000 cells were seeded in 10 mL of DMEM per flask 17 h before transfection. Transfections were performed using 10 μg of total DNA per flask with a 1:1:1:1 ratio of vectors coding for mGlu2-A258TAA-C2KKXX, FLAG-SNAP-mGlu2-A248TAG-C1KKXX, pIRE4-PGK-ePrFRS and pNEU-hMbPylRS­(AF)-4xU6M15 in 500 μL of JetPrime buffer. A total of 20 μL of JetPrime Reagent per flask was added and incubated for 25 min at RT. Last, the transfection mixtures were added to the cells and incubated at 37 °C for 6 h. After 6h, medium was replaced with 10 mL fresh DMEM supplemented with 0.2 mM TCOK and 0.3 mM PrF. The medium was further exchanged with fresh TCOK and PrF-supplemented medium after 24 and 48 h. 72 h after transfection SNAP-tag labeling was performed on adherent cells at 37 °C and 5% CO_2_ for 2 h using a final concentration of 300 nM SNAP-surface 488 and excess dye was removed by three cycles of washing with DPBS without Ca^2+^ and Mg^2+^ at RT. TCOK labeling was performed right after SNAP-tag labeling using a final concentration of 1 μM Cy3B tetrazine in acquisition buffer incubated for 15 min at 37 °C. Excess dye was removed by three cycles of washing with DPBS without Ca^2+^ and Mg^2+^ at RT. PrF labeling was performed last using a final concentration of 10 μM AF647-picolyl-azide, 1.5 mM Aminoguanidine, 1.98 mM BTTAA, 0.36 mM CuS0_4_, 2 mM Na-Ascorbate in acquisition buffer incubated for 15 min at 37 °C. Excess dye was removed by three cycles of washing with DPBS without Ca^2+^ and Mg^2+^ at RT. Crude membrane fractions were prepared as describe above.

Receptors were solubilized using 100 μL of acquisition buffer containing 1% LMNG (w/v) and 0.1% CHS Tris (w/v) per membrane fraction (corresponding to cells cultured in one T-75 flask) for 30 min on ice. Subsequently, the solubilization mixture was centrifuged for 20 min at 4 °C and 21,000*g*, and the supernatant was mixed with 900 μL of acquisition buffer containing 0.11% GDN (w/v). The diluted sample was then loaded onto a 200 μL anti-FLAG resin gravity column equilibrated with acquisition buffer and incubated at 4 °C for 1 h, then reapplied 2 times. The column was washed with 1 mL acquisition buffer containing 0.01% LMNG (w/v), 0.001% CHS Tris (w/v) and 0.01% GDN (w/v), then 1 mL acquisition buffer containing 0.005% LMNG (w/v), 0.0005% CHS Tris (w/v) and 0.005% GDN (w/v) and finally eluted with three times 120 μL acquisition buffer containing 0.005% LMNG (w/v), 0.0005% CHS Tris (w/v), 0.005% GDN (w/v) and 0.2 mg/mL FLAG peptide (Sigma). For acquisition, samples were diluted in acquisition buffer to a final concentration of 0.0025% LMNG (w/v), 0.00025% CHS Tris (w/v), 0.0025% GDN (w/v) in the absence or presence of 10 mM l-glutamate ± 10 μM 3′-[[(2-Cyclopentyl-2,3-dihydro-6,7-dimethyl-1-oxo-1H-inden-5-yl)­oxy]­methyl]-[1,1′-biphenyl]-4-carboxylic acid (BINA, Tocris).

### Single Molecule FRET Acquisition

2-color smFRET experiments with pulsed interleaved excitation (PIE) and multiparameter fluorescence detection (MFD) were performed on a home-built confocal microscope (Figure S1) using the SPCM 9.85 software (B&H) as described previously.^28^ Modifications are described in the following. A combination of 530/20 (530AF20, Omega Optical, Brattleboro, VT, USA) and 530/10 nm (FLH532- 10, Thorlabs, Maisons-Laffitte, France) bandpass filters were used for Cy3B excitation. A 488/10 (Z488/10 X, Chroma, Bellows Falls, VT, USA) bandpass filter was used for SNAP-surface488 excitation. A 635/10 (FLH635-10, Thorlabs, Maisons-Laffitte, France) bandpass filter was used for AF647 excitation. The excitation beam was polarized using a polarizing beam splitter and laser powers used at the entrance to the microscope body where set to 25 μW for blue (488 nm) and 12 μW for red (635 nm) or 30 μW for green (535 nm) and 12 μW for red (635 nm). Inside the microscope, the light was reflected by dichroic mirrors that match the excitation/emission wavelengths of the respective fluorophore combinations (Cy3B/AF647: FF545/650-Di01, Semrock, Rochester, NY, USA and SNAP-surface488/AF647: FF500/646-Di01, Semrock, Rochester, NY, USA) and coupled into a 100×, numerical aperture 1.4 objective (Nikon, France). The emitted photons where split by polarization and the following emission filters were used: Cy3B parallel and perpendicular ET BP 585/65 (Chroma, Bellows Falls, VT, USA); AF647 parallel and perpendicular FF01-698/70-25 (Semrock, Rochester, NY, USA); AF488 parallel 535/50 BrightLine HC, perpendicular 530/43 BrightLine HC (Semrock, Rochester, NY, USA). Dual color emission was separated using FF649LP long pass filters (parallel and perpendicular, Semrock, Rochester, NY, USA) for Cy3B with AF647 and AT608LP (parallel, Chroma, Bellows Falls, VT, USA) together with FF560LP (perpendicular, Semrock, Rochester, NY, USA) for SNAP-surface488 with AF647.

3-Color smFRET measurements were performed on a home-built confocal setup with PIE and MFD as described previously (Figure S2).^7^ Briefly, the triple labeled mGlu samples were diluted to 10–20 pM and measured for 1–3 h in solution. The laser powers used during the experiments were 40 μW for blue (485 nm), 30 μW for green (565 nm) and 15 μW for red (647 nm) lasers. The collected data were analyzed with the open source software package PIE Analysis with MATLAB (PAM).^29^ Bursts were selected as described previously using a sliding time window approach on the total signal, requiring at least 8 photons per time window of 500 μs and at least 40 photons in total per burst.^30^ Triple-labeled bursts were selected based on stoichiometry thresholds (*S*_BG_, *S*_BR_, and *S*_GR_) using a lower boundary of 0.3 and an upper boundary of 0.8. To additionally remove photobleaching and blinking events, the ALEX-2CDE filter^31^ was applied, which was calculated pairwise for the three excitation channels. The 3-color PDA analysis was done as described before.^7^ Detected fluorescence intensities from all three fluorophores were used to calculate the uncorrected FRET efficiency (proximity ratios, PR) values. To determine the underlying populations for each PR histogram, a maximum likelihood estimator based on a three component Gaussian mixture was used. First, the PR_GR_ histogram is fitted with a binomial distribution function. Then, the two- and three-dimensional description of the 3-color FRET data is done by binomial and trinomial distributions. For the apo state, a single state (*R*_O_) was used to described both the PR_GR_ and PR_BR_ histograms. In the presence of glutamine, both PR_GR_ and PR_BR_ histograms were fitted with contributions from 2 states (*A*_C_ and *I*_Glu_).

## Results

To investigate potential correlations between the different domains, we developed a triple labeling strategy based on orthogonal, cotranslational incorporation of two distinct reactive ncAAs using two distinct stop codons together with fusion to a genetically encoded self-labeling SNAP tag.^32^ In the first protomer of mGlu2, a SNAP-tag is fused to the N-terminus and we incorporated a p-propargyloxy-l-phenylalanine (PrF) in response to the Amber codon (TAG) at position 248 within the lower lobe (Figure 2a). In the second protomer, no SNAP tag was used and a trans-cyclooct-2-en-l-lysine (TCOK) was incorporated in response to an Ochre codon (TAA) at position 258 within the lower lobe. The 258 position was chosen to improve protein yields due to an inefficient incorporation of TCOK in response to TAA at the 248 position (Figure S3). PrF and TCOK were incorporated by coexpression of the receptor genes harboring the respective premature stop codons with an engineered B. stearothermophilus tyrosyl-tRNA_CUA_/E. coli tyrosyl-tRNA synthetase pair (PrFRS-tRNA_CUA_)^20^ and an engineered M15 pyrolysyl-tRNA_UUA_/M. barkeri pyrolysyl-tRNA synthetase pair (PylRS-tRNA_UUA_)^27^ in HEK293T cells, respectively (Figure 2a). To specifically label dimers with a single set of these three functionalities, we employed our previously described C1/C2 system derived from the γ-Aminobutyric acid B receptor (GABA_B_) quality control system.^20,25^ Subsequent labeling was performed directly on living mammalian cells using commercially available reagents for (i) the O^6^-alkylguanine-DNA-alkyltransferase activity of the SNAP tag (Figure 2b, D1 = Atto488), (ii) SPIEDAC (Figure 2b, A1/D2 = Cy3B), and (iii) CuAAC (Figure 2b, A2 = AF647). Of note, this approach is not compromised by natively present reactive amino acid functionalities and does not require postexpression protein processing before dye conjugation. However, it has been shown that CuAAC and SPIEDAC cannot be performed in a single one-pot reaction,^33^ which is why we performed them sequentially with an intermediate washing step.

**Figure 2:**
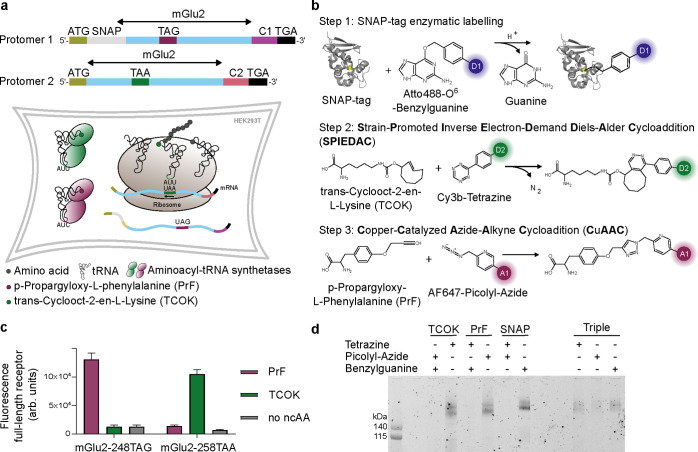
Orthogonal triple labeling strategy. (a) Top: Schematic representation of the mGlu2 protomer genes expressed to produce the 3-color smFRET sensor. Important features of the protomers are highlighted, including ATG start codons, SNAP tag, premature TAG and TAA stop codons within the mGlu2 gene, the GABA_B_ C1 and C2 tails and the final TGA stop codons. Bottom: Schematic representation of cotranslational double ncAA incorporation in HEK cells. (b) Three-step site-specific labeling reactions of mGlu2 receptors in the membrane of living cells prior to solubilization: (1) SNAP-tag labeling using Atto488–0^6^-benzylguanine; (2) TCOK labeling using Cy3B-tetrazine; (3) PrF labeling using AF647-picolyl-azide. (c) Orthogonal suppression of TAG and TAA stop codons. Fluorescence signal of HEK cells expressing the mGlu2 receptor bearing either a TAG or a TAA premature stop codon and an N-terminal SNAP fusion protein, labeled at the cell surface with Lumi4-Tb-BG. Only full-length receptors expressed through successful ncAA incorporation are presented at the cell-surface and labeled. All expressions were performed with both synthetase/tRNA pairs in the absence or presence of either ncAA. Data are given as the mean ± standard deviation from biological triplicates. (d) Evaluation of the labeling specificity. Receptors with incorporated TCOK, PrF or SNAP fusion protein were subjected to the respective labeling reactions as indicated with (+) using Cy3B-tetrazine, AF546-picoly zide and/or Cy3B-benzylguanine and detected by in-gel fluorescence after solubilization and SDS-PAGE.

First, we verified the orthogonality of the two aminoacyl-tRNA synthetase/tRNA pairs to incorporate PrF in response to TAG suppression at position 248 in combination with TAA suppression for TCOK incorporation at position 258 within the lower lobes (Figure 2c). tRNA wobble pairing has previously been described for the combination of TAG and TAA incorporation^15^ and would lead to the subsequent attachment of dyes at undesired positions. To exclude this possibility, we took advantage of the fact that termination at each of the premature stop codons, located within the extracellular domains, does not result in translation of the full-length receptors. Hence, only full-length receptors that comprise the 7TM domains, resulting from incorporation of ncAA, will be contranslationally inserted into the membrane of the endoplasmic reticulum and trafficked to the plasma membrane of cells. Truncated VFT domain fragments, resulting from translation termination, will not be inserted into the plasma membrane. Therefore, specific labeling of the upper lobes of the VFT domains of cell surface-presented full-length mGlu2 receptors via N-terminal SNAP-tag labeling using the membrane impermeable Lumi4-Tb-BG occurs only upon suppression of premature stop codons. This served as a reporter for proper ncAA incorporation and demonstrated the required specificity of a TAG template for PrF- and a TAA template for TCOK-mediate full-length receptor expression, while neither tRNA wobbling nor a strong read-through in the absence of ncAA was observed (Figure 2c).

Next, we addressed the specificity of dye conjugation to the two ncAAs and the SNAP-tag (Figure 2d). Cross-reactivity leading to attachment of dyes to positions other than the desired ones would compromise the specificity of the labeling and thereby complicate assignment of observed FRET distributions to the conformational states of interest. Therefore, we expressed receptor genes harboring (1) a premature TAA at position 258 (Figure 2d, TCOK), (2) a premature TAG at position 248 (Figure 2d, PrF), (3) no premature stop codon but an N-terminal SNAP-tag (Figure 2d, SNAP) and (4) a combination of one protomer with a SNAP-tag as well as a premature TAG at position 248 and one protomer with a premature TAA at position 258 to compose our anticipated 3-color sensor (Figure 2d, Triple). In all cases, the two tRNA/synthetase pairs were coexpressed and both ncAAs were included in the growth media. We then performed either a single or a 2-step double-labeling and detected fluorescently labeled receptors in-gel after solubilization. The results demonstrated that TCOK is specifically labeled with the tetrazine dye but not with the picolyl-azide and benzylguanine dyes (Figure 2d, TCOK), PrF is specifically labeled with the picolyl-azide dye but not the tetrazine and the benzylguanine dyes (Figure 2d, PrF) while the SNAP-containing receptor is specifically labeled with the benzylguanine dye but not the tetrazine and picolyl-azide dyes (Figure 2d, SNAP). Thus, the two ncAAs are specifically incorporated only in response to their respective premature stop codons and the three reactions are highly selective, leading to perfect orthogonality of our triple labeling approach. Furthermore, weak but detectable bands confirmed the successful labeling of the triple functionalized receptors for each of the three orthogonal reactions (Figure 2d, Triple).

We then characterized two double-labeled sensors monitoring either the VFT closure (o → c, Figure 3a) or lower lobe reorientation (R → A, Figure 3b) via 2-color smFRET experiments. For each sample, two of the three positions were labeled using the same positions, strategies, and dyes as were used for the subsequent 3-color sensor. The results of the 2-color FRET experiments suggested a nearly complete closure of the VFT domains by a transition from a single low FRET (Figure 3a, Apo, gray) to a medium FRET population (Figure 3a, Glutamate, yellow) but only a partial stabilization of the fully reoriented active state at saturating glutamate concentrations (Figure 3b). These findings are in very good agreement with our previous observations with similar, but distinct FRET sensors. They suggest the existence of an intermediate population in the presence of glutamate, which is in dynamic equilibrium between the resting and active orientations at time scales slower than the ∼3 ms observation time of our smFRET approach.^20^

**Figure 3:**
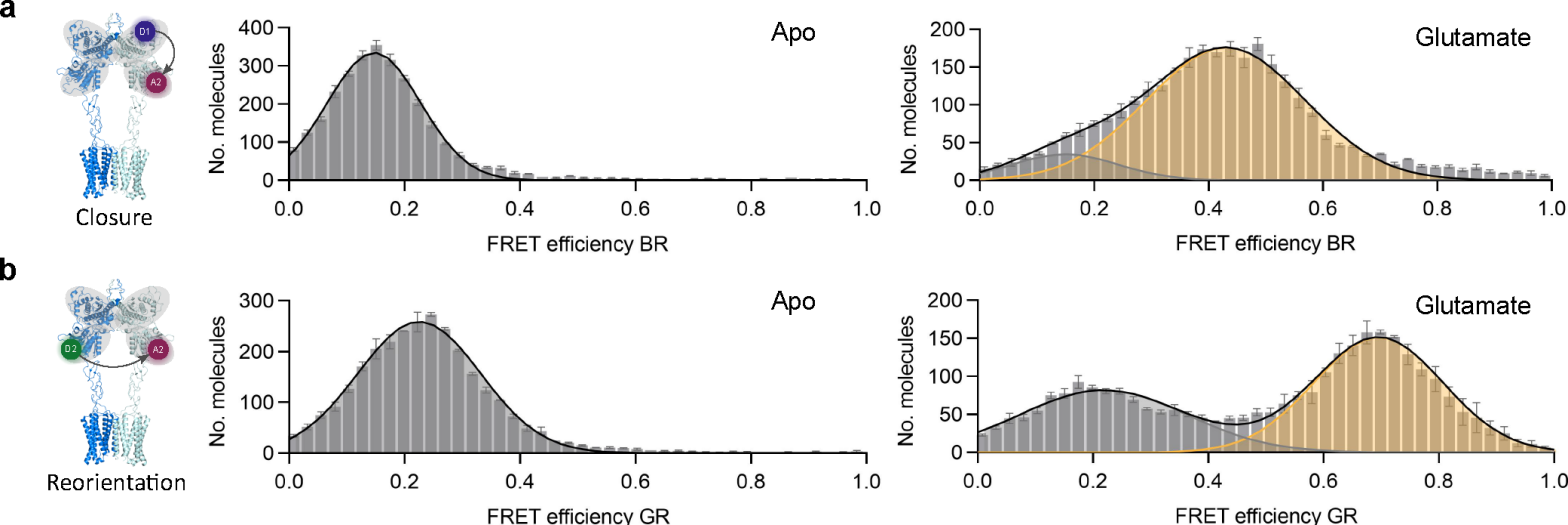
2-Color smFRET experiments suggest efficient VFT domain closure but incomplete stabilization of the reoriented state. Histograms of VFT domain closure (a) and reorientation (b) sensors in the apo condition and in the presence of a saturating glutamate concentration (10 mM). The closure sensor (a) was labeled with Atto488-O^6^-benzylguanine via the SNAP-tag on the upper lobe and with AF647-picolyl-azide via PrF incorporated in response to a TAG stop codon at position 248 within the lower lobe. Sensors bearing only a single labeled protomer were achieved using the engineered GABA_B_ receptor quality control system. The reorientation sensor (b) was obtained by labeling of PrF incorporated in response to a TAG stop codon at position 248 within the lower lobe of protomer 1 with AF647-picolyl-azide and TCOK incorporated in response to a TAA stop codon at position 258 within the lower lobe of protomer 2 with Cy3B-tetrazine. The engineered GABAB receptor quality control system was used to specifically label the desired dimer populations on the cell surface. Data are given as the mean ± standard deviation from biological triplicates.

Next, we measured our triple-labeled sensor using 3-color smFRET (Figures 4a,b and S4) to directly correlate VFT closure (o → c, FRET efficiency BR) and reorientation (R → A, FRET efficiency GR) on individual single molecules. As expected, in the absence of ligand, a single state (*R*_O_) was observed with low FRET population for both, VFT domain closure (BR FRET pair, *E*_BR_ = 0.2) and reorientation (GR FRET pair, *E*_GR_ = 0.12, Figure 4a). In the presence of glutamate, the R → A sensor revealed the two expected conformations corresponding to the resting and active orientations with similar relative populations (55 vs 45% respectively, Figure 4b, FRET efficiency GR). Likewise, the o → c sensor revealed two populations (Figure 4b, FRET efficiency BR), with the high FRET state corresponding to the closed VFT domains in the active orientation. The 3-color smFRET data allowed us to separate these two species and determine their individual FRET efficiencies with BR, reporting on the closure of the VFT domain. For the active species (high FRET efficiency GR), the FRET efficiency BR corresponded to the closed VFT domain as expected (*E*_BR_ = 0.46). However, to our surprise, for the species remaining in the resting state (low FRET efficiency GR), we found a FRET efficiency BR that appeared intermediate between those of the open and the closed conformation (*E*_BR_ = 0.34 vs *E*_BR,open_ = 0.2 and *E*_BR,closed_ = 0.46). A similar finding was obtained by plotting the diagonal FRET efficiency BG between the upper lobe of one VFT and the lower lobe of the other but with a less well-defined FRET efficiency BR for the two states in the presence of glutamate (Figure S5).

**Figure 4:**
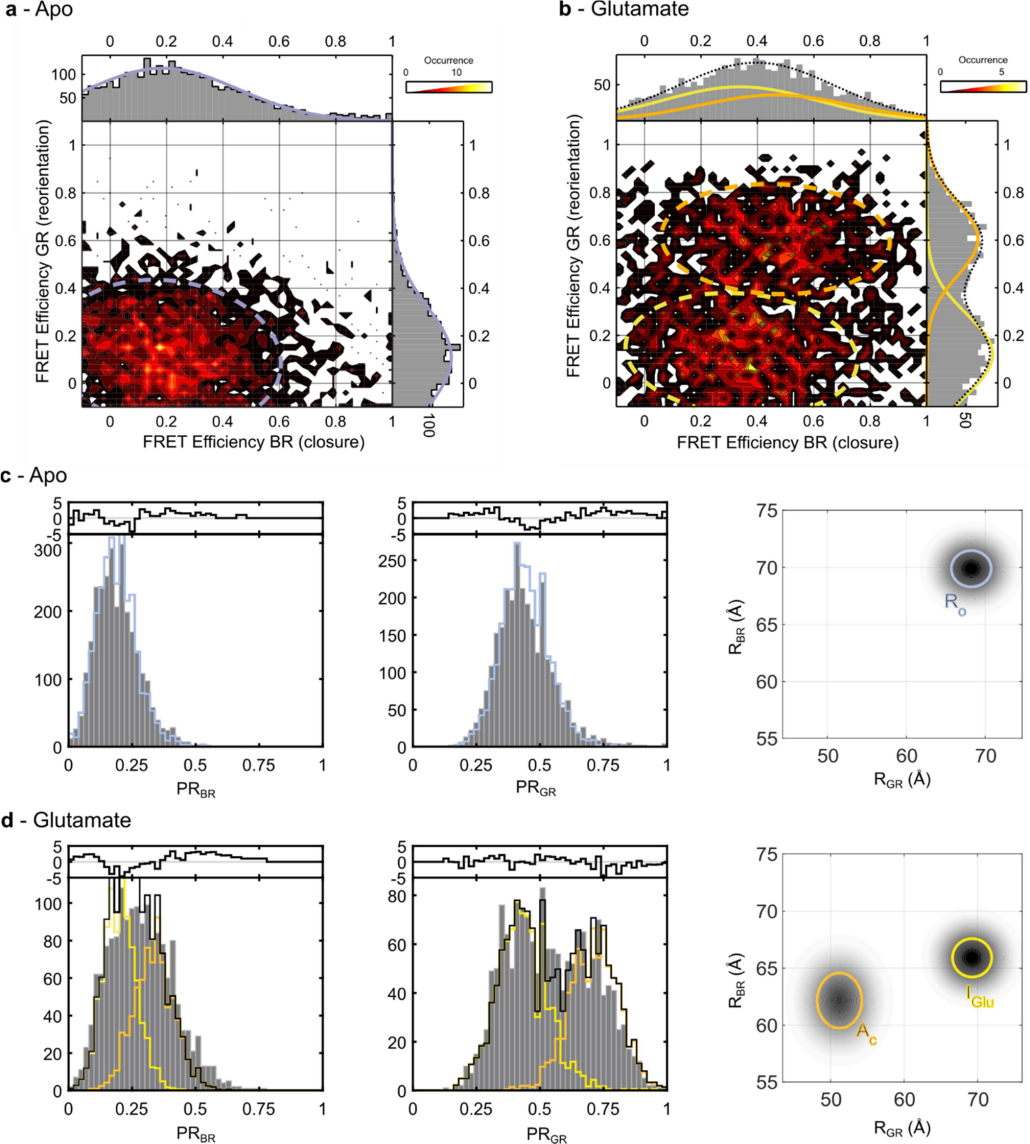
Three-color smFRET experiments unveil coordinated rearrangements of the VFT domains of mGlu2 and an unknown conformational intermediate. (a, b) Two-dimensional projections of VFT domain closure (FRET efficiency BR) versus reorientation (FRET efficiency GR) in the absence (a) and presence of a saturating glutamate concentration (b). (c) One-dimensional projections of the proximity ratio for VFT domain closure (PRBR, left) and reorientation (PRGR, middle) and two-dimensional apparent distance distribution histogram (right) extracted from PDA analysis for the apo state. (d) One-dimensional projections of the proximity ratio for VFT domain closure (PRBR, left) and reorientation (PRGR, middle) and two-dimensional apparent distance distribution histogram (right) extracted from PDA analysis under the influence of a saturating glutamate concentration (10 mM). Two populations are sufficient to describe the 3-color data, which is clearly observable in the PRGR projection (d, middle) but more difficult to detect for the PRBR data (d, left). However, the PDA analysis reveals a clear reduction in the distance between upper and lower lobes (PRBR) between the apo (c, right) and glutamate condition (d, right) indicating a slight closure of the VFTs. Shown is a representative data set. A second data set can be found in Figure S4.

To quantitatively analyze the 3-color smFRET data, we performed our previously established 3-color photon distribution analysis (3C-PDA) using either a 1 or 2-state model to reinforce the idea that this shift corresponds to a change in the interdye distance distribution (Figures 4c,d, S6 and S7).^7^ Consistent with the 2-color data (Figure 3a,b), the 1-dimensional projection of the o → c sensor (PR_BR_) demonstrated the expected shift from a low FRET value (Figure 4c, left panel) to a higher FRET value in the presence of glutamate (Figure 4d, left panel), indicative of VFT closure. Accordingly, the 1-dimensional projection of the R → A sensor revealed a single low FRET state (Figure 4c, middle panel) in the apo condition, which, in the presence of glutamate, splits into two populations (Figure 4d, middle panel). Thanks to the 3-color approach, we found that only two species were sufficient to describe the data in the presence of glutamate and we were able to separate out two conformations in the PR_BR_ histogram (Figure 4d, left panel). The population observed with higher PR_GR_ values can be assigned to the A_C_ state. It has a corresponding shift in the PR_BR_ value indicative of full VFT domain closure, which corresponds to a shorter distance *R*_BR_ between the upper and lower lobes (Figure 4d, orange population in right panel). The conformation with the lower PR_GR_ value corresponds to an increased distance between the lower lobes and has an intermediate PR_BR_ value, indicative of a slightly reduced distance *R*_BR_ between the upper and lower lobes (Figure 4d, yellow in right panel). This distance is slightly shorter than the one obtained for the *R*_O_ state, measured under the apo condition (Figure 4c, blue in right panel). This implies that the glutamate-bound VFT domains in the resting orientation adopt a slightly closed conformation that is intermediate between the fully open state observed in the apo condition (*R*_O_) and the glutamate-induced fully closed state observed for the receptors in the reoriented conformation (*A*_C_). Instead of establishing a dynamic equilibrium between the *R*_C_ and *A*_C_ conformations, as we formerly postulated,^20^ the 3-color data clearly show that glutamate-induced activation of mGlu2 receptors involves a previously unknown intermediate state we term *I*_Glu_, which exists in equilibrium with the *A*_C_ state.

On the basis of the identification of the *I*_Glu_ intermediate state, we reanalyzed the 2-color data obtained for the VFT domain closure sensor using the properties of this intermediate state (Figure 5a). We determined the FRET efficiency of the fully closed state (*E*_BR,closed_ = 0.45) using a saturating concentration of glutamate and the positive allosteric modulator BINA (Figure S8), which stabilizes the fully active state.^20^ We then refitted the histogram of the 2-color VFT domain closure sensor in the presence of glutamate using a two Gaussian fit model by constraining a 55%/45% ratio between the low FRET and high FRET populations, respectively (corresponding to the resting/active reorientation ratio determined from the FRET efficiency GR distribution from 3-color measurements shown in Figure 4b), and an *E*_BR,closed_ value of 0.45 for the high FRET population (Figure S8a). In doing so, we obtained an *E*_BR,intermediate_ value of 0.27 for the low FRET state (Figure 5a), which is higher than measured in the 2-color experiments of the apo state (*E*_BR,open_ = 0.14, Figure 3a). Similarly, we performed a 2-color smFRET experiment on a new VFT domain closure sensor, where the SNAP-tag has been removed and both upper and lower lobes were labeled using ncAAs (Figure 5b). TCOK was incorporated in response to a TAA at position 358 in the upper lobe and PrF in response to a TAG codon at position 248 in the lower lobe into the same mGlu2 protomer and labeled with Atto488-tetrazine and AF647-picoly-azide, respectively. Fixing the ratio of the resting/active reorientation to 55%/45% and the high FRET value to that measured with glutamate and the allosteric modulator BINA as above, we again obtained an intermediate population (*E*_BR,intermediate_ = 0.37) with a value between what is observed in the apo state and in the fully closed state (*E*_BR,open_ = 0.18 and *E*_BR,closed_ = 0.50, Figure S8b). This alternative fitting approach describes the data very well (as given by the black dashed line in Figure 5) and further supports the existence of an intermediate state, in which the VFT domain is partially closed. These results could not have been conclusively determined from 2-color FRET measurements alone.

**Figure 5:**
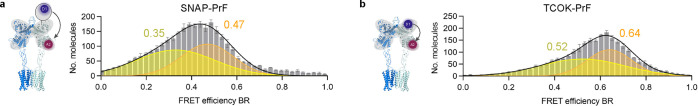
Constrained analysis of 2-color smFRET data on the VFT domain closure recovers the intermediate state. (a, b) SmFRET histograms from 2-color experiments in the presence of 10 mM glutamate are shown for the SNAP-PrF VFT closure sensor (a, corresponds to data in Figure 3a) and the closure sensor obtained through incorporation and labeling of TCOK at position 358 with Atto488-tetrazine and PrF at position 248 with AF647-pycolyl-azide within the same protomer using the GABAB quality control system (b). The high FRET values, corresponding to the fully closed state, were fixed to 0.47 (a) and 0.64 (b), respectively, as obtained from control measurements performed in the presence of glutamate and allosteric modulator BINA (see Figure S3). The ratio between high and low FRET states were constrained to 55%/45% as obtained from 3-color measurements (see Figure 4b). The low FRET values extracted from this procedure were found to be intermediate between the fully closed, high FRET values and the low FRET values obtained for the apo state (see Figure S3). Data are given as the mean ± standard deviation from biological triplicates.

## Discussion

In this study, we present a triple labeling strategy that allows the attachment of three distinct organic dyes to proteins through a combination of two different reactive ncAAs and a self-labeling SNAP-tag. We demonstrate that the incorporation of ncAAs mediated by two different tRNA/synthetase pairs in mammalian HEK293T cells occurs in an orthogonal manner, each in response to its designated premature stop codon. Furthermore, the three bioorthogonal labeling reactions occur with high specificity leading to a single triple-labeled product suitable for 3-color smFRET studies. While we incorporated a single ncAA into each protomer of the dimeric mGlu2 receptor and screened different positions within the lower lobe to achieve efficient TAA reassignment with TCOK, we additionally show that incorporation of the two ncAAs into a single protomer is feasible (Figure 5b). Thus, our approach is not limited to the labeling of a single ncAA per subunit within protein complexes but can also be applied to label 2 positions within monomeric proteins. An inherent drawback of stop codon suppression arises from the overall reduction in protein yields, which may benefit from determining suitable positions that show good ncAA incorporation, especially when multiple ncAAs are used. However, for applications requiring minimal sample amounts, such as smFRET, this is not detrimental and may partially be compensated for by a simple expression scale up. More important for smFRET is the need for efficient labeling with small organic dyes, as low triple labeling would require long acquisitions times to collect sufficient data. As these are typically collected at room temperature, one has to ensure that the sample is sufficiently stable over the time course of the acquisition, which we previously achieved through a careful optimization of detergent conditions for the mGlu2 receptor.^20,34^ To achieve efficient triple labeling, we combined enzymatic SNAP-tag labeling with the CuAAC and the SPIEDAC “click” chemistries, all three being among the fastest and most selective bioorthogonal reactions available.^35^ Moreover, we chose these reactions as all components (tRNA/synthetase vectors, ncAAs and reactive dyes) are easily accessible to others without the need for special equipment and expertise in organic chemistry. While our example demonstrates that CuAAC can be performed under optimized conditions without impacting the functionality of receptors after extraction from cells, other ncAAs and chemistries orthogonal to the SPIEDAC reaction such as SPAAC and Staudinger ligation with incorporated azide moieties can likely be optimized to circumvent the use of copper as a catalyst. However, these will likely result in less efficient labeling due to the partial reduction of azides in mammalian cells^36^ and the slower kinetics of these reactions.^35^ Thus, albeit triple ncAA incorporation has been demonstrated recently in mammalian cells, triple labeling using three distinct ncAAs likely still requires careful optimization of expression and labeling conditions to be applicable to smFRET. Accordingly, the classical SNAP-tag represents a versatile tool to attach the third dye as it is genetically encoded and provides a high labeling efficiency,^32^ which may even be further improved through the use of SNAPf^37^ and SNAP-tag2.^38^ A limitation of the SNAP-tag technology arises from its size of ∼19 kDa, which is generally only applicable for N- or C-terminal labeling and may have an impact on native protein function and dynamics. Thus, careful controls have to be included if it is to be used to derive structural and dynamics information as we did previously for the mGlu2 receptors based on pharmacological assays^39^ and ligand-induced conformational changes.^20,34^ However, its size can be an advantage as it might allow to improve separation of dyes to perform measurements within the optimal dynamic range for a given FRET pair, thereby providing access to the study of smaller proteins than our example herein.

Thanks to this triple labeling strategy, we were able to monitor the glutamate-induced closure of the VFT domains and their reorientation simultaneously for each single molecule using 3-color smFRET on a home-built (Figure 5) as well as a commercially available setup (Figure S4) and describe a previously unknown intermediate state during mGlu2 activation. Additionally, the 3-color PDA analysis performed on the data obtained by the two different smFRET setups revealed similar results and confirmed our findings on the intermediate state of the mGlu receptor (Figures 4c,d, S4c,d, S6 and S7). Two structural studies of the mGlu5 receptor recently proposed the presence of an intermediate state where both VFT domains are closed,^40,41^ which we have previously postulated for the mGlu2 receptor.^20^ However, based on the data presented here, this state seems not to be significantly populated by the mGlu2 receptor under equilibrium conditions in a carefully selected detergent micelle environment. We previously demonstrated the functional allosteric modulation and G protein-coupling of the mGlu2 receptor in these micelles.^34^ An intermediate state has also been proposed for the mGlu2 receptor with one VFT domain in the open and the other in the closed conformation but with the lower lobes being separated as seen in the resting state.^24,42^ However, if such a conformation was significantly populated and stable in our measurements, this would be reflected by the presence of two, well-defined FRET populations for the resting state, provided transitions between the open and closed conformations occur on time scales slower than the observation time of the fluorescent bursts (∼3 ms). Alternatively, submillisecond VFT domain oscillations would lead to an average FRET efficiency found at intermediate values between the fully open and fully closed states. Such rapid dynamics would be expected to impair state resolution when performing single-particle reconstructions using cryo-EM. In addition, we have no evidence in our 3-color smFRET experiments and 3-color PDA analysis describes the data well using only two populations (an intermediate, partially closed state (*I*_Glu_) and the fully closed (*A*_C_) state).

## Conclusions

In summary, we demonstrated the discovery of a previously unknown intermediate state of the mGlu2 receptor when activated by its natural full agonist glutamate, which was obscured in previous classical 2-color smFRET experiments. This discovery was made possible only by the 2-dimensional resolution of 3-color smFRET experiments. To perform these experiments on such a multidomain, multimeric, human neuroreceptor, it was necessary to establish a new and sophisticated orthogonal triple-labeling strategy to overcome previous limitations related to the use of cysteine-maleimide labeling. Our strategy, based on expression in mammalian cells, now opens the possibility to perform triple-labeling on a broader range of proteins and complexes without being limited by natively present reactive amino acids. This opens up the possibility to study other mGlu receptor homo- but also heterodimers as well as other class C GPCRs and multimeric membrane proteins to elucidate symmetric and asymmetric structural features.^43^ Such experiments will provide deeper insights into how conformational states and dynamics regulate protein function through concerted structural rearrangements.

## Supplementary Material



## Notes

Microscopy schemes, additional data on ncAA incorporation and smFRET, and summary of used plasmids (PDF)

## Abbreviations

7TM: seven transmembrane domain
A: active receptor orientation
BR: blue to red energy transfer
c: closed VFT domain
CRD: cysteine-rich domaine
*E*: FRET efficiency
GAGA_B_: γ-aminobutyric acid B receptor
GPCR: G protein-coupled receptor
GR: green to red energy transfer
*I*_Glu_: glutamate-induced intermediate state
mGlu receptor: metabotropic glutamate receptor
ncAA: noncanonical amino acid
o: open VFT domain
PR: proximity ratio
PrF: p-propargyloxy-l-phenylalanine
PrFRS-tRNA_CUA_: orthogonal tRNA/synthetase pair used for PrF incorporation in response to TAG stop codon
PylRS-tRNA_UUA_: orthogonal tRNA/synthetase pair used for TCOK incorporation in response to TAA stop codon
R: resting receptor orientation
smFRET: single molecule Förster resonance energy transfer
TAA: ochre stop codon
TAG: amber stop codon
TCOK: trans-cyclooct-2-en-l-lysine
VFT: Venus flytrap
