# Supplementary material for: Triple Labeling Resolves a GPCR Intermediate State by Using Three-Color Single Molecule FRET
Source: J Am Chem Soc. 2025 May 15;147(21):17689–700. doi: 10.1021/jacs.4c18364 (PMC12123625; doi:10.1021/jacs.4c18364)
Supplement: Supplementary file 1 [file ja4c18364_si_001.pdf]

Supplementary material

Triple labeling resolves a GPCR intermediate state by using three-color single molecule FRET

Leo Bonhomme[1], Ecenaz Bilgen[2], Caroline Clerté[1], Jean-Philippe Pin[3], Philippe Rondard[3], Emmanuel Margeat[1], Don C. Lamb[2*], Robert B. Quast[1*]

[1]Centre de Biologie Structurale (CBS), Univ. Montpellier, CNRS, INSERM, Montpellier, 34090 France

[2]Department of Chemistry and Center for Nanoscience, Ludwig-Maximilians-Universität München (LMU), Munich, 81377 Germany

[3]Institut de Génomique Fonctionnelle (IGF), Univ. Montpellier, CNRS, INSERM, Montpellier, 34090 France

[*]Corresponding authors

Table of Contents

Figure S1: Confocal 2-color MFD-PIE setup. 3
Figure S2: Confocal 3-color MFD-PIE setup. 4
Figure S3: Incorporation efficiency of TCOK in response to TAA suppression at different positions within the lower lobe of the VFT domain. 5
Figure S4: 3-color smFRET data obtained on a commercial Luminosa setup from PicoQuant. 6
Figure S5: Blue-Green FRET Efficiencies. 8
Figure S6: 3-color PDA analysis of the 3-color mGlu sensor in the apo state 9
Figure S7: 3-color PDA analysis of the 3-color mGlu sensor in the presence of saturating glutamate concentration (10 mM). 10
Figure S8: Data used for the determination of constrains needed for reanalysis of the 2-color VFT domain closure sensor. 11
Table S1: Summary of expressed receptor variants. 12

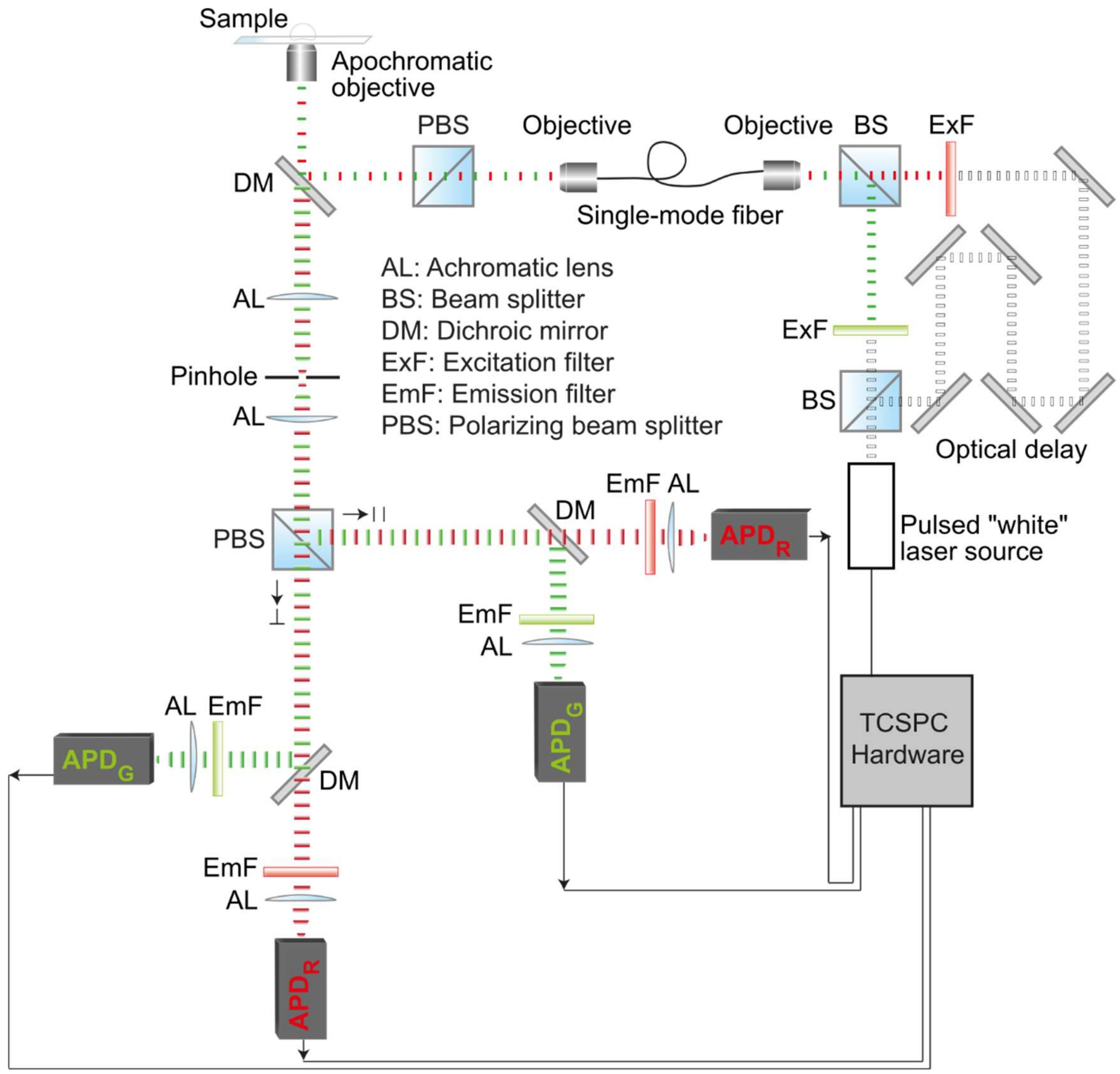

Figure S1: Confocal 2-color MFD-PIE setup. Details about the setup can be found in the experimental section. In brief, a picosecond pulsed "white" laser is split into two beams via a beam splitter (BS). The straight beam is filtered for excitation of the donor (either blue or green), while the reflected beam is optically delayed by ~25 ns through several mirrors and then filtered for excitation of the acceptor (red). The two interleaved pulses are then recombined through a beam splitter, overlapped by coupling through a single-mode fiber and then collimated. The beam is then polarized before exciting the sample and emission is collected in parallel and perpendicular polarization pathways, each equipped with two single-photon counting devices, one for donor and one for acceptor emission.

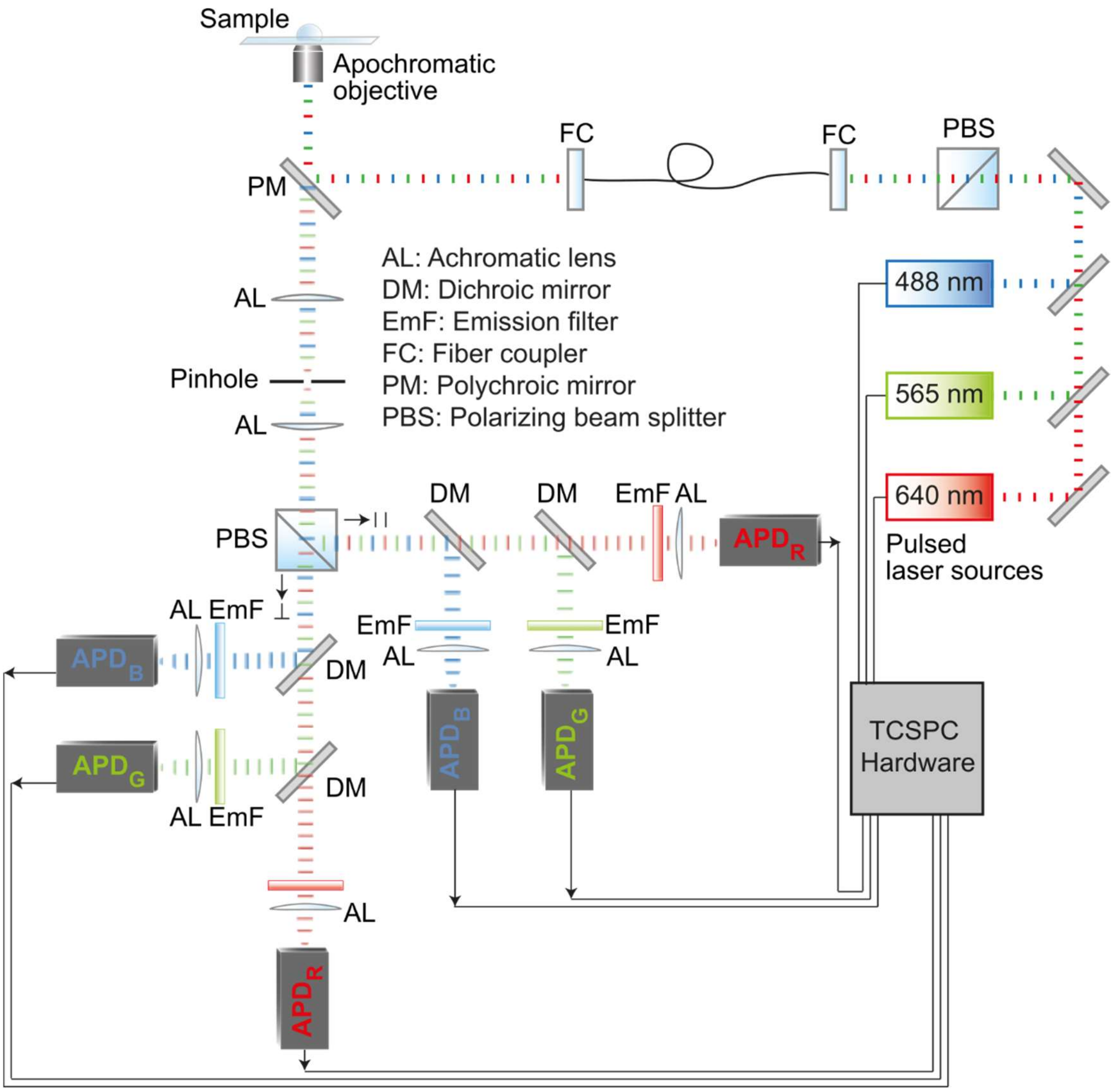

Figure S2: Confocal 3-color MFD-PIE setup. Details on the setup can be found in the experimental section. In brief, three picosecond pulsed lasers are interleaved, polarized, overlapped by coupling into a single-mode fiber and collimated before exciting the sample. The parallel and perpendicular emission channels are collected on three, single-photon counting devices, one for each color (i.e. blue = donor 1, green = acceptor 1/donor 2 and 3 = acceptor 2).

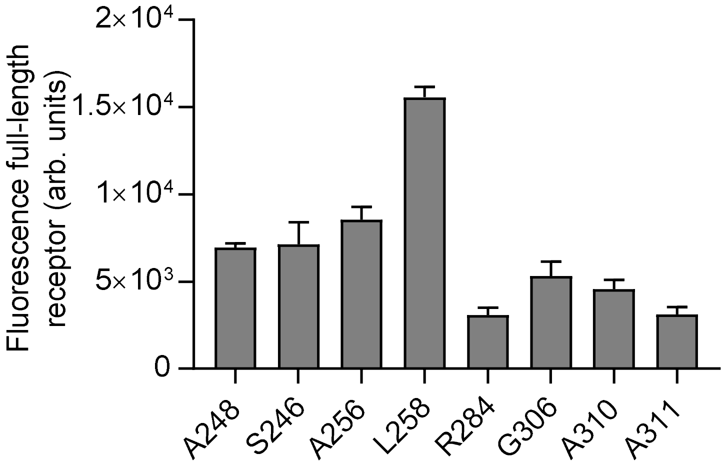

Figure S3: Incorporation efficiency of TCOK in response to TAA suppression at different positions within the lower lobe of the VFT domain. Incorporation was evaluated based on total fluorescence measured from HEK293T cells expressing the indicated stop codon mutants in the presence of PylRS-$tRNA_{UUA}$ and TCOK. Labeling of full-length receptors, presented at the cell surface as a result of successful incorporation of TCOK, was achieved through N-terminal SNAP-tag labeling using the cell-impermeable Lumi4-Tb-SNAP substrate. Data are shown as the mean +/- standard deviation from three biological replicates.

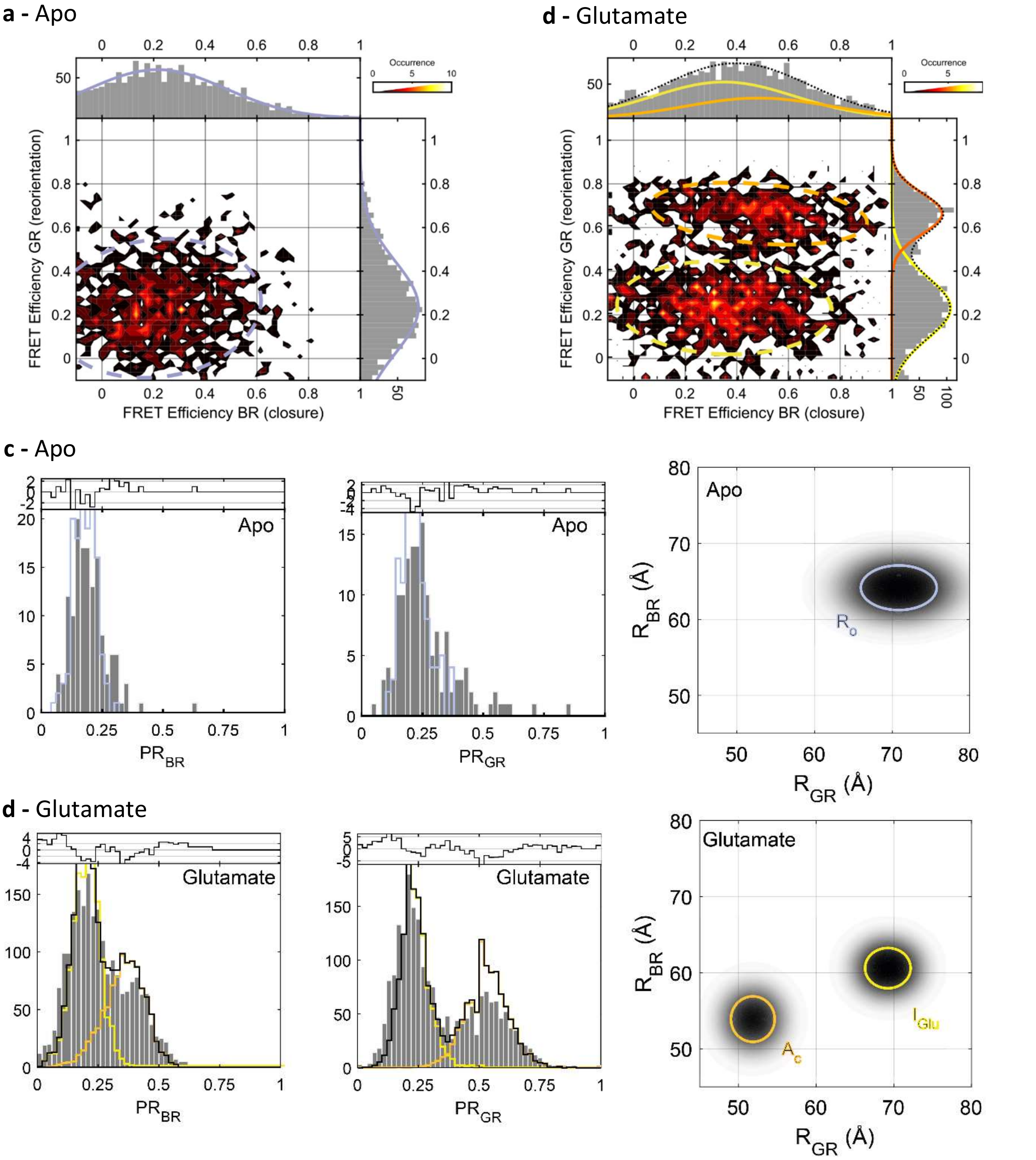

Figure S4: 3-color smFRET data obtained on a commercial Luminosa setup from PicoQuant. a-b) Two-dimensional FRET histograms of the FRET efficiency GR (VFT reorientation) versus the FRET efficiency BR (VFT closure) of our 3-color sensor are shown in the absence (a, Apo) and presence of 10 mM saturating glutamate concentration (b, Glutamate). **c**) One-dimensional projections of the proximity ratio for VFT domain closure (PR_{BR}, left) and reorientation (PR_{GR}, middle) and two-dimensional apparent distance distribution histogram (right) extracted from a PDA analysis for the apo state. **d**) One-dimensional projections of the proximity ratio for VFT domain closure (PR_{BR}, left) and reorientation (PR_{GR}, middle) and two-dimensional apparent distance distribution histogram (right) extracted from a PDA analysis under the influence of a saturating glutamate concentration (10 mM). The data was obtained on a commercial Luminosa microscope in the lab of P. Tinnefeld (LMU Munich, Germany). The setup configuration of the Luminosa microscope used was similar to the home-built 3-color MFD-PIE setup (see Figure S2). The fluorescent molecules were excited by pulsed lasers at 485 nm, 530 nm and 640 nm wavelength, operated at 13.3 MHz. The 532 nm and 640 nm laser pulses were delayed by 25 ns and 50 ns respectively to the 485 nm laser pulse. The laser light was guided into

the epi-illuminated confocal microscope by a quad-band beam splitter (QB405/485/530/640) and focused by a water immersion objective (60x, NA 1.2) into the sample. The emitted fluorescence was collected through the objective and spatially filtered by a pinhole with a 100 µm diameter. The fluorescence signal was spectrally split into three detection channels by a long-pass beam-splitter (LP532) followed by a second long-pass beamsplitter (LP635). The fluorescence was cleaned by bandpass filters in each detection channel (485 nm excitation: BP511/20), (530 nm excitation: BP582/64), (640 nm excitation: BP690/70), and focused on single-photon counting modules.

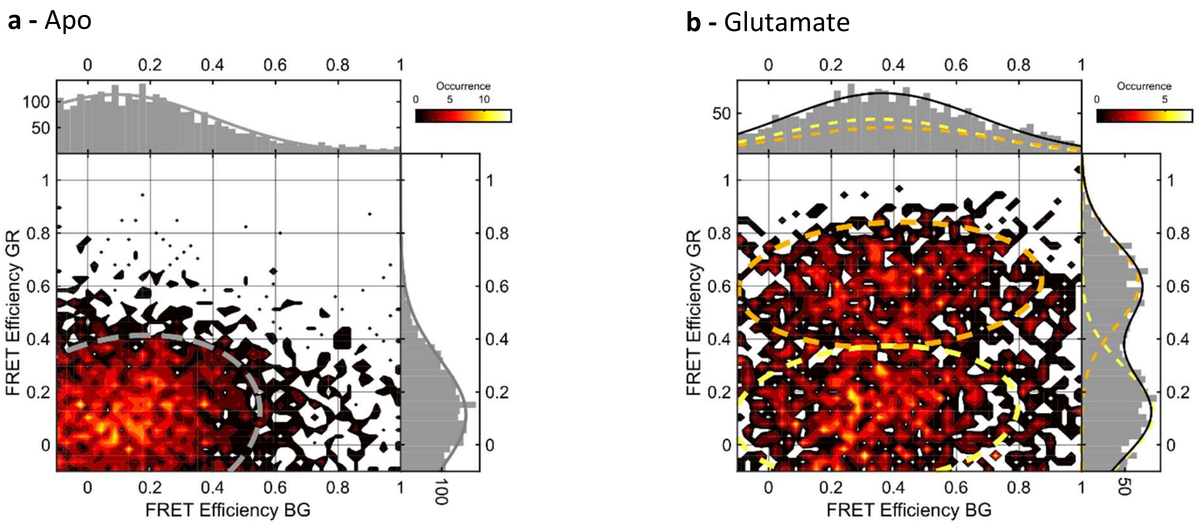

Figure S5: Blue-Green FRET Efficiencies. a-b) Two-dimensional FRET efficiency plots of the lower lobe (FRET efficiency GR) and diagonal (FRET efficiency BG) VFT domain sensors in the absence (**a**) and presence of glutamate (**b**) extracted from the 3-color smFRET acquisition. The FRET efficiency BG reports on the distance between the N-terminal SNAP-tag in one protomer and position 258 of the lower-lobe of the other protomer. Hence, it is a convolution of the conformation of the VFT domain and the orientation of the domains.

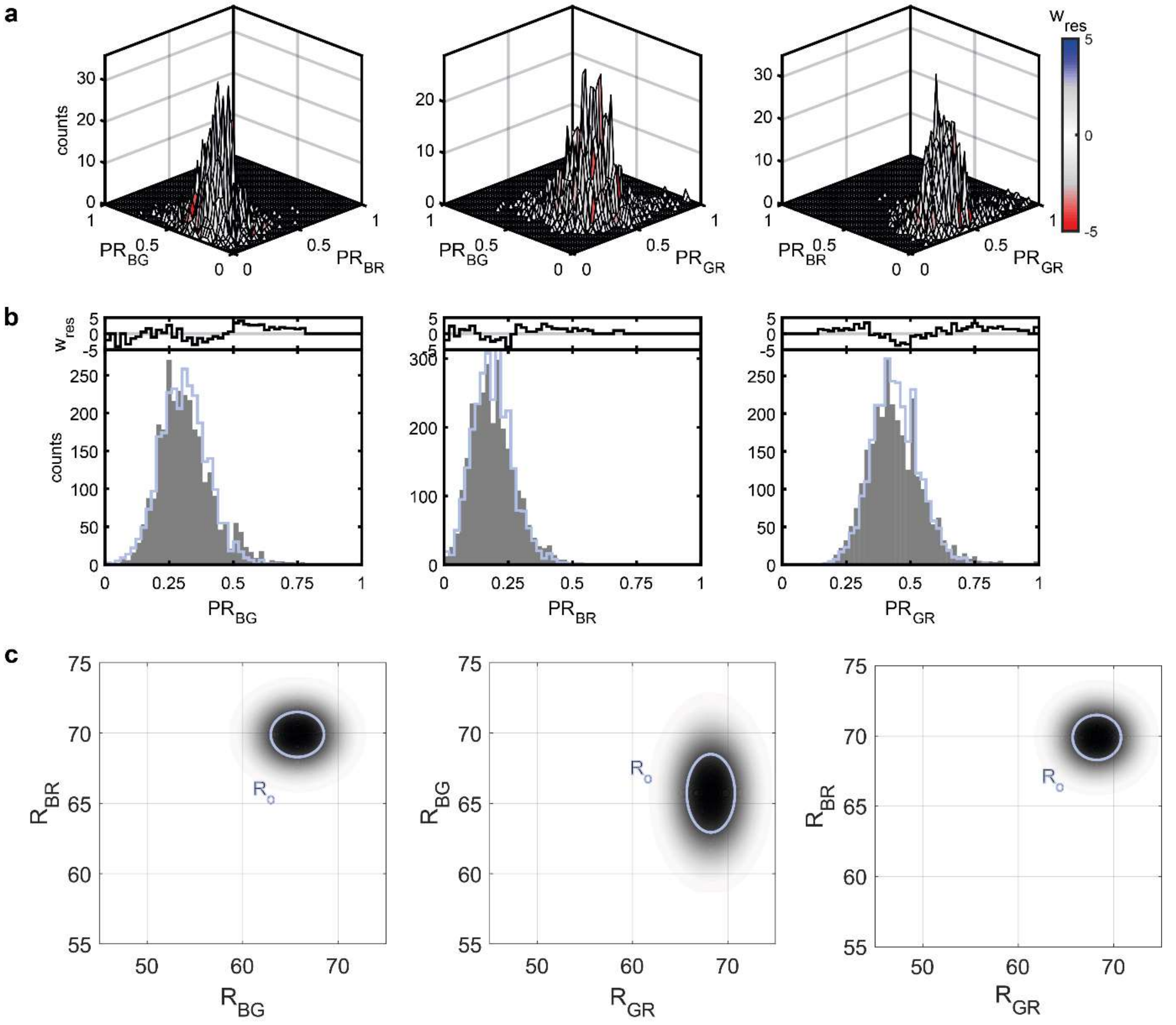

Figure S6: 3-color PDA analysis of the 3-color mGlu sensor in the apo state. a) Two-dimensional plots showing correlations between the proximity ratios extracted from the data for all three dye pairs collected on the 3-color MFD-PIE setup: PR_{BG}-PR_{BR} (left), PR_{BG}-PR_{GR} (middle) and PR_{BR}- PR_{GR} (right). **b**) One-dimensional projections of the proximity ratio for the distance change between the lower lobes and VFT domain (PR_{BG}, right), VFT domain closure (PR_{BR}, middle) and reorientation (PR_{GR}, left). **c**) Two-dimensional apparent distance distribution histograms extracted from PDA analysis for the proximity ratios PR_{BG}-PR_{BR} (left), PR_{BG}-PR_{GR} (middle) and PR_{BR}- PR_{GR} (right).

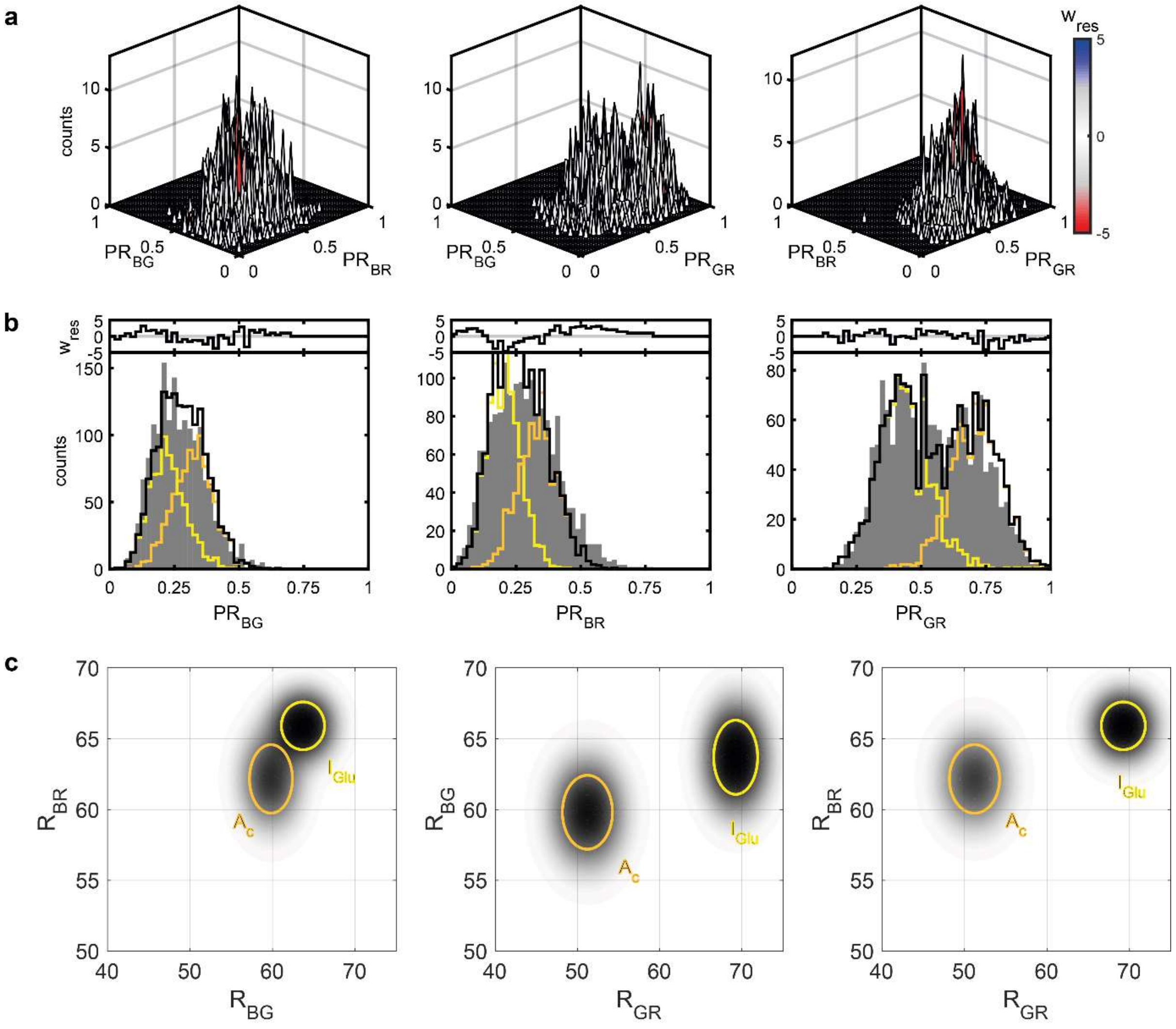

Figure S7: 3-color PDA analysis of the 3-color mGlu sensor in the presence of saturating glutamate concentration (10 mM). a) Two-dimensional plots showing correlations between the proximity ratios extracted from the data for all three dye pairs collected on the 3-color MFD-PIE setup: PR_{BG}-PR_{BR} (left), PR_{BG}-PR_{GR} (middle) and PR_{BR}- PR_{GR} (right). Two populations are visible in the data. **b**) One-dimensional projections of the proximity ratio for the distance change between the lower lobes and VFT domain (PR_{BG}, right), VFT domain closure (PR_{BR}, middle) and reorientation (PR_{GR}, left). **c**) Two-dimensional apparent distance distribution histograms extracted from PDA analysis for the proximity ratios PR_{BG}-PR_{BR} (left), PR_{BG}-PR_{GR} (middle) and PR_{BR}- PR_{GR} (right) showing the two detected populations.

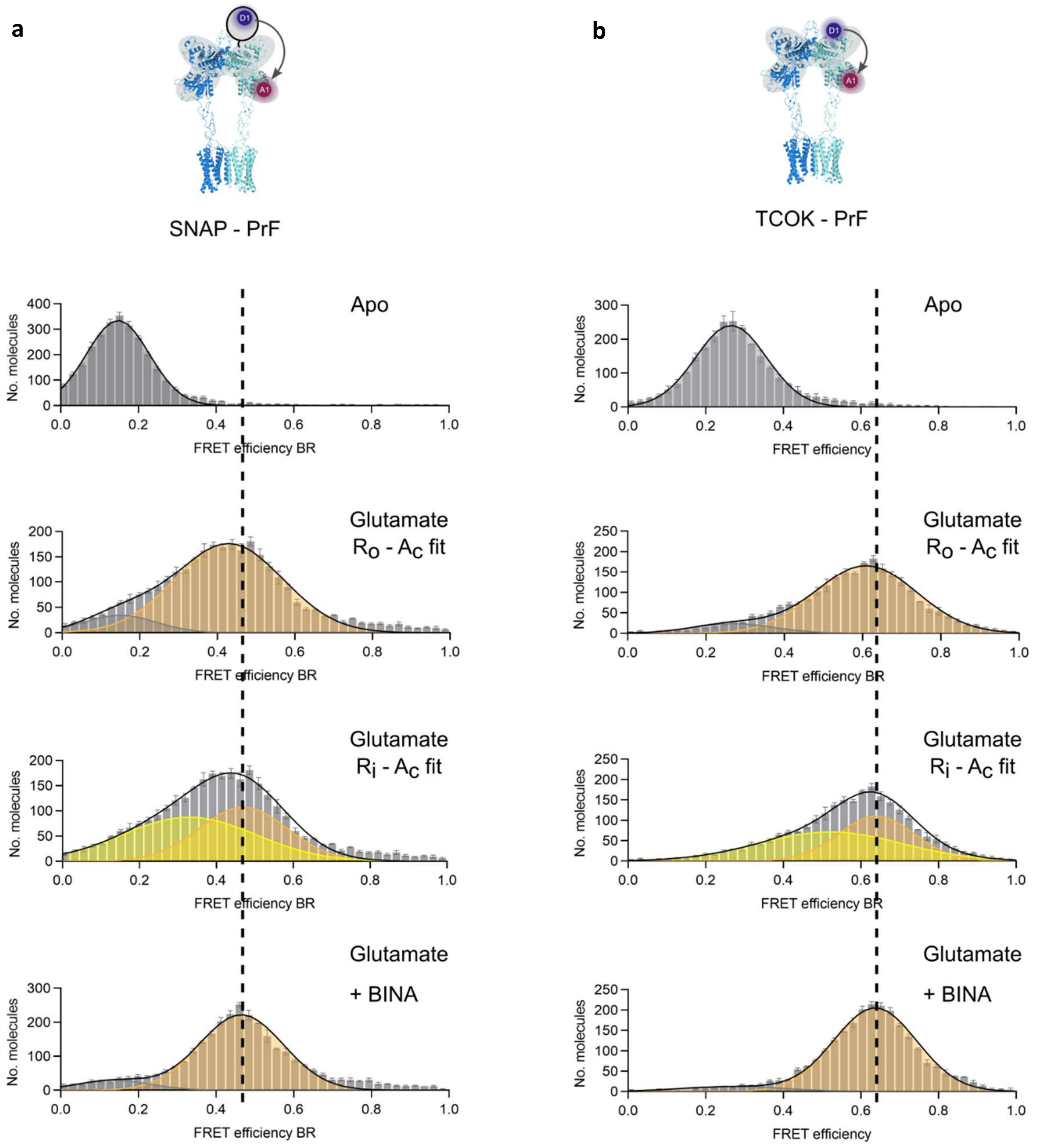

Figure S8: Data used for the determination of constrains needed for reanalysis of the 2-color VFT domain closure sensor. a-b) Two-color smFRET histograms are shown for the SNAP-PrF closure sensor (**a**) and the orientation sensor established by incorporation of PrF and TCOK (**b**). Data are shown for the apo condition and in the presence of a saturation concentration of glutamate (10 mM) or glutamate + allosteric modulator BINA (10 mM + 10 µM, respectively). Histograms were fit with a single Gaussian (apo), two Gaussians with variable center and amplitude values (glutamate Ro – Ac fit and glutamate + BINA) or two Gaussians with fixed amplitudes (55% low FRET and 45% high FRET) and fixed high FRET center value. Data are given as the mean +/- standard deviation from biological triplicates.

Table S1: Summary of expressed receptor variants.

Name	SNAP-tag	Mutations	C-terminus	Reference
FLAG-SNAP-mGlu2-A248TAA	yes	A248TAA	WT	Figure S3
FLAG-SNAP-mGlu2-S246TAA	yes	S246TAA	WT	Figure S3
FLAG-SNAP-mGlu2-A256TAA	yes	A256TAA	WT	Figure S3
FLAG-SNAP-mGlu2-L258TAA	yes	L258TAA	WT	Figure S3, 2c
FLAG-SNAP-mGlu2-R284TAA	yes	R284TAA	WT	Figure S3
FLAG-SNAP-mGlu2-G306TAA	yes	G306TAA	WT	Figure S3
FLAG-SNAP-mGlu2-A310TAA	yes	A310TAA	WT	Figure S3
FLAG-SNAP-mGlu2-A311TAA	yes	A311TAA	WT	Figure S3
FLAG-SNAP-mGlu2-A248TAG	yes	A248TAA	WT	Figure 2c
mGlu2-C2KKXX	no	none	C2KKXX	Figure 2d, 3a, 5, S8
mGlu2-A248TAG-C1KKXX	no	A248TAG	C1KKXX	Figure 2d, 3b
mGlu2-C1KKXX	no	none	C1KKXX	Figure 2d
mGlu2-L258TAA-C2KKXX	no	L258TAA	C2KKXX	Figure 2d, 3b, 4, S4, S5, S6, S7
FLAG-SNAP-mGlu2-C1KKXX	yes	none	C1KKXX	Figure 2d
FLAG-SNAP-mGlu2-A248TAG-C1KKXX	yes	none	C1KKXX	Figure 2d, 4, 5a, S4, S5, S6, S7, S8a
mGlu2-A248TAG-R358TAA-C1KKXX	no	A248TAG R358TAA	C1KKXX	Figure 5b, S8b
